# The Hidden Iceberg of ADPKD: Early Organomegaly-Driven Malnutrition and Sarcopenia Beyond Preserved eGFR

**DOI:** 10.3390/ijms27041667

**Published:** 2026-02-09

**Authors:** Matteo Brambilla Pisoni, Martina Catania, Rodolfo Fernando Rivera, Liliana Italia De Rosa, Kristiana Kola, Michele Paolisi, Pierpaolo Bianca, Sara Farinone, Micaela Petrone, Lorena Citterio, Giuseppe Vezzoli, Maria Teresa Sciarrone Alibrandi

**Affiliations:** 1 OU Nephrology and Dialysis, IRCCS San Raffaele Scientific Institute, 20132 Milan, Italyvezzoli.giuseppe@hsr.it (G.V.);; 2 School of Specialization in Nephrology, Università Vita Salute San Raffaele, 20132 Milan, Italy; catania.martina@hsr.it (M.C.); paolisi.michele@hsr.it (M.P.);; 3 OU Nephrology and Dialysis, Pio XI Hospital ASST-Brianza, 20832 Desio, Italy; 4 Clinical and Health Psychology Unit, IRCCS San Raffaele Hospital, 20132 Milan, Italy; farinone.sara@hsr.it; 5 OU Obstretics and Ginecology, IRCCS San Raffaele Scientific Institute, 20132 Milan, Italy; 6 Genomics of Renal Disease and Hypertension Unit, IRCCS San Raffaele Scientific Institute, 20132 Milan, Italy

**Keywords:** autosomal dominant polycystic kidney disease (ADPKD), organomegaly, malnutrition, sarcopenia, body composition, bioelectrical impedance analysis (BIA), extracellular water, polycystic liver disease, htTKLV, nutritional assessment

## Abstract

Autosomal dominant polycystic kidney disease (ADPKD) is the most frequent monogenic kidney disease (≈4 cases per 10.000 inhabitants) and a major cause of end-stage kidney disease (ESKD). Beyond progressive cystic enlargement of the kidneys and frequent extrarenal involvement, adults with ADPKD often exhibit a distinctive “body phenotype” with central adiposity and marked abdominal distension due to renal and hepatic organomegaly. In this setting, conventional anthropometric indices such as body mass index (BMI) and crude body weight are of limited value, as they cannot distinguish nutritional tissues (muscle, subcutaneous fat) from non-nutritional mass (cyst fluid, fibrotic tissue, or expanded extracellular water). This review summarizes the current evidence on malnutrition and sarcopenia in adult ADPKD, with a focus on the impact of organomegaly and adiposity. Cross-sectional work using the modified Subjective Global Assessment (SGA) has shown that approximately one-third of ambulatory ADPKD patients are at risk of becoming, or have become, malnourished, and that height-adjusted total kidney and liver volume (htTKLV) is the strongest clinical predictor of malnutrition, whereas eGFR plays a secondary role. Bioelectrical impedance analysis (BIA) further demonstrates a disease-specific body composition phenotype, with increased total and extracellular body water, particularly in the trunk, a reduced phase angle and reduced lean mass, consistent with early malnutrition and sarcopenia. These alterations are present even at relatively preserved kidney function and, in matched analyses, distinguish ADPKD from non-ADPKD CKD. Prospective data from a multicenter cohort indicate that the baseline SGA-defined nutritional status independently predicts short-term eGFR decline in typical ADPKD, supporting malnutrition as a potential modifier of renal trajectory rather than a mere correlate of advanced disease. In parallel, narrative syntheses on adiposity highlight that a higher BMI, waist circumference and visceral fat are associated with larger total kidney volume, faster eGFR loss and greater symptom burden, and raise concern for a sarcopenic obesity phenotype in which excess fat and cystic mass coexist with low muscle mass. Collectively, these findings support a pathophysiological model in which organomegaly-driven mechanical effects (early satiety, gastrointestinal discomfort), systemic inflammation, insulin resistance and cyst-related metabolic reprogramming converge to produce “hidden malnutrition” in ADPKD, masked by apparent overweight. From a clinical perspective, malnutrition and sarcopenia should be regarded as central, disease-modifying components of the ADPKD phenotype. Routine nutritional screening (e.g., SGA/PG-SGA) and BIA-based body composition assessment, particularly in patients with severe organomegaly or symptomatic polycystic liver disease, should be integrated into ADPKD care pathways, and individualized, muscle-preserving nutritional strategies should be tested in future prospective studies.

## 1. Introduction

Autosomal dominant polycystic kidney disease (ADPKD) is the most frequent monogenic kidney disease. Historically, its prevalence was often quoted in the range of 1:400–1:1000 in the general population; however, more recent population-based and registry-based studies have provided more conservative and internally consistent estimates. In large European cohorts, point prevalences derived from administrative databases and direct screening range from approximately 2.4 to 3.9 per 10,000, with screening-adjusted estimates of 3.3–4.6 per 10,000, supporting an overall prevalence of about four cases per 10,000 inhabitants, i.e., below the European Union threshold for a rare disease [1,2].

### 1.1. Genetics and Disease Mechanisms

Autosomal dominant polycystic kidney disease (ADPKD) is caused predominantly by heterozygous pathogenic variants in PKD1 (≈80–85% of cases) and PKD2 (≈15%), encoding polycystin-1 and polycystin-2, respectively. These proteins co-localize in the primary cilium and other cellular compartments, where they act as mechanosensors and regulators of multiple signaling pathways, including Ca^2+^ homeostasis, cAMP signaling, mTOR activity, planar cell polarity, proliferation and apoptosis. The classical “two-hit” model posits that focal loss of the normal allele in a subset of tubular epithelial cells leads to clonal cyst formation, while additional mechanisms such as haploinsufficiency, dosage effects and genetic modifiers are thought to modulate vascular and extrarenal phenotypes. Clinically, PKD1 variants are associated with earlier onset and more severe kidney disease (mean age at ESKD ~54 years), whereas PKD2 variants usually confer milder disease and later ESKD (mean ~74 years) [3,4,5].

In addition to PKD1 and PKD2, a growing number of “minor” ADPKD-spectrum genes have been recognized, including GANAB, DNAJB11, ALG8, ALG9, HNF1B, NEK8, and, more recently, monoallelic loss-of-function variants in IFT140 [6,7,8,9,10].

Among the expanding group of ADPKD-spectrum genes, those with the most robust and clinically relevant association with adult ADPKD-like phenotypes include GANAB, DNAJB11 and IFT140.

GANAB encodes the α-subunit of glucosidase II and its heterozygous variants cause a relatively mild form of autosomal dominant polycystic kidney and liver disease, probably through impaired maturation and trafficking of polycystin-1 and -2 [6].

DNAJB11- and glycosylation defect-related disease (e.g., ALG8, ALG9) typically present with atypical ADPKD-like phenotypes, with small or normal-sized kidneys, few cysts and interstitial fibrosis [7].

Monoallelic IFT140 variants, originally linked to recessive ciliopathies, have recently emerged as the third most common genetic cause of an ADPKD-spectrum phenotype, usually characterized by bilateral kidney cysts, relatively mild and atypical imaging features, and later onset of kidney function decline compared with classic PKD1-associated disease [9,11].

### 1.2. Renal Manifestations of Autosomal Dominant Polycystic Kidney Disease

Renal disease in ADPKD is characterized by progressive development and enlargement of multiple renal cysts arising from 1 to 2% of nephrons but eventually occupying most of the parenchyma [12,13].

Renal complications include macrohematuria, intrarenal cyst hemorrhage, cyst infection, nephrolithiasis, and chronic flank or abdominal pain. Functional manifestations are characterized by tubular concentrating defects, low-grade proteinuria or albuminuria, and a slow but relentless progression from chronic kidney disease (CKD) to end-stage kidney disease (ESKD) [13].

Total kidney volume (TKV), especially when indexed to height (htTKV), is now established as a key prognostic biomarker and a surrogate endpoint in clinical trials [14].

MRI-based Mayo imaging classes stratify patients into slow vs. rapid progressors according to age-adjusted TKV [15].

### 1.3. Extrarenal Manifestations

ADPKD is a multisystem disorder. Systemic arterial hypertension represents one of its earliest and most prevalent manifestations, typically developing in early adulthood, often one to two decades before any measurable decline in eGFR, and affecting the majority of patients by mid-life. Hypertension in ADPKD is thought to result from intrarenal ischemia and early activation of the renin–angiotensin–aldosterone system, compounded by endothelial dysfunction and increased arterial stiffness, and is a major driver of left ventricular hypertrophy and cardiovascular morbidity [12].

The most frequent extrarenal structural manifestation is autosomal dominant polycystic liver disease (ADPLD/PLD), with liver cysts detectable in up to 80–94% of ADPKD patients over 35 years [13], particularly in women and in the setting of cumulative estrogen exposure [16]. Hepatic cysts arise from bile duct epithelium and can lead to massive hepatomegaly, with total liver volumes exceeding several liters in advanced cases. Additional extrarenal features include intracranial aneurysms and other arterial aneurysms or dissections, cardiac valvular abnormalities, colonic diverticulosis, abdominal wall hernias, and pancreatic or seminal vesicle cysts [12].

Taken together, the renal, cardiovascular and extrarenal manifestations underline that ADPKD is a complex, systemic disorder, and that optimal care requires a coordinated, multidisciplinary approach involving nephrologists, hepatologists and cardiologists throughout the disease course [17].

### 1.4. Metabolic Abnormalities in ADPKD

Beyond the structural renal and extrarenal abnormalities, ADPKD is increasingly recognized as a disorder of profound metabolic dysregulation. Polycystin deficiency triggers fundamental metabolic reprogramming at the cellular level, characterized by a shift from oxidative phosphorylation toward aerobic glycolysis (Warburg effect), mitochondrial dysfunction, accumulation of reactive oxygen species, and chronic activation of pro-inflammatory pathways [18,19]. These alterations are not merely consequences of cyst expansion but appear causative in the pathogenesis of cyst growth itself [18].

At the systemic level, adults with ADPKD exhibit a constellation of metabolic derangements including insulin resistance, impaired glucose tolerance, dyslipidemia, and dysregulated AMPK-mTOR signaling—features that overlap with metabolic syndrome but appear distinct in their mechanistic origins [19]. Mitochondrial dysfunction and oxidative stress contribute to endothelial dysfunction, enhanced inflammatory tone, and activation of fibrogenic pathways, thereby linking metabolic abnormalities to both renal disease progression and cardiovascular risk [18,19].

Importantly, these metabolic disturbances are detectable even in patients with preserved eGFR, suggesting that ADPKD-associated metabolic dysfunction begins early and may contribute independently to disease trajectory [19].

### 1.5. The “Body Phenotype” of Adult ADPKD

Several epidemiologic series and recent reviews [13,20] highlight that adults with ADPKD often present with:

Overweight or obesity (elevated BMI);

Increased waist circumference and central adiposity;

Progressive abdominal distension from kidney and liver enlargement;

Reduced physical activity due to pain, fatigue, abdominal mass effect, and comorbidities [21].

This combination creates a quite distinctive body phenotype: a protuberant abdomen driven by a mixture of visceral fat and cystic organ volume, sometimes accompanying relatively thin extremities. To the non-specialist eye, and even to many nephrologists, such patients often appear “well nourished” or “too well nourished”. However, conventional anthropometric indices such as BMI and body weight do not distinguish between:

- Nutritional tissues (skeletal muscle, subcutaneous fat, energy stores), and
- Non-nutritional mass (cyst fluid, fibrotic tissue, expanded extracellular water).

This mismatch is the conceptual starting point for understanding why malnutrition risk is systematically underestimated in ADPKD.

In the following sections, we focus specifically on the nutritional status, body composition and sarcopenia in adult ADPKD, with particular emphasis on the impact of renal and hepatic organomegaly, and we summarize the emerging evidence that links this “hidden iceberg” to clinical outcomes and disease progression.

**Figure 1:**
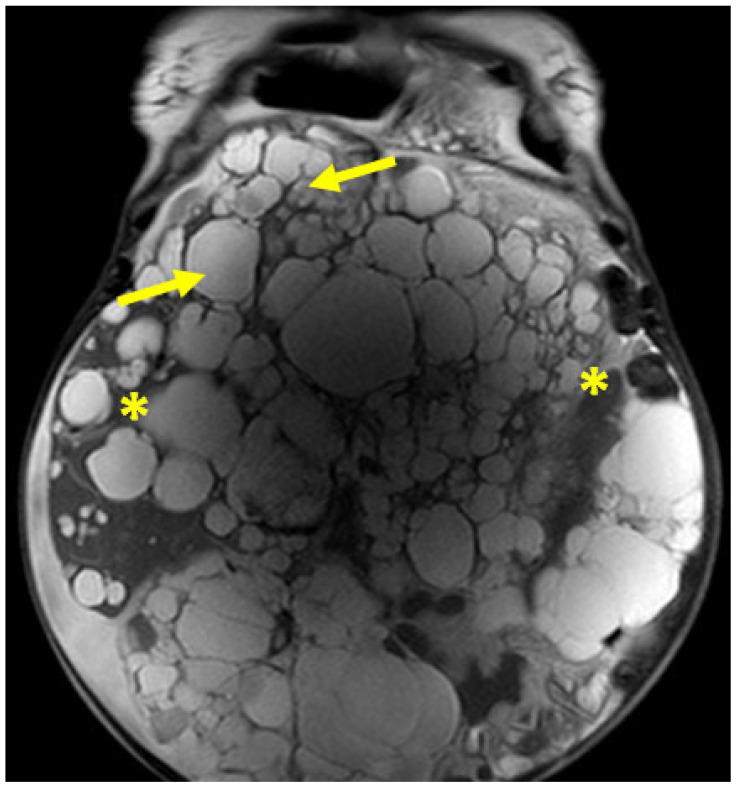
ADPKD body phenotype. Representative MRI scan demonstrating marked organomegaly in ADPKD. Axial MRI image showing bilateral massively enlarged polycystic kidneys (asterisks) and multiple hepatic cysts (arrows), characteristic of advanced ADPKD with severe organomegaly. Note the displacement of adjacent structures and significant reduction in available abdominal cavity space for other organs.

Given the mounting evidence of malnutrition in ADPKD and its potential clinical implications, we conducted a comprehensive literature review to synthesize current knowledge on this topic. Figure 1 provides an overview of the pathophysiological pathway linking organomegaly to malnutrition, sarcopenia, and adverse outcomes.

## 2. Methods

### 2.1. Literature Search Strategy

A comprehensive literature search was conducted in PubMed/MEDLINE, Embase, Web of Science, and the Cochrane Library from inception through December 2024 to identify studies addressing nutritional status, body composition, malnutrition, sarcopenia, and clinical outcomes in adult ADPKD patients. The search combined MeSH terms and keywords for ADPKD (including PKD1/PKD2), nutritional parameters (malnutrition, protein-energy wasting, body composition, bioelectrical impedance, phase angle, sarcopenia, muscle mass), and clinical variables (organomegaly, kidney/liver volumes, htTKV, htTKLV, anthropometry). Manual screening of reference lists supplemented database searches. No language restrictions were applied.

### 2.2. Study Selection and Inclusion Criteria

Eligible studies included adult patients (≥18 years) with confirmed ADPKD, reported nutritional or body composition data, provided quantitative outcomes (malnutrition prevalence, phase angle, muscle indices, associations with organ volume or renal function), and were original research (observational studies, trials, cohorts). Exclusion criteria: pediatric populations, case reports (<10 patients), conference abstracts, animal studies, and articles addressing exclusively genetic mechanisms without clinical nutritional data. Two reviewers independently screened titles and abstracts; disagreements were resolved by consensus.

### 2.3. Data Extraction and Quality Assessment

Extracted data included study design, sample characteristics (age, sex, CKD stage), nutritional assessment methods (SGA, BIA, imaging-derived indices), organ volumes (htTKV, htTKLV), and outcomes (eGFR trajectory, hospitalization, quality of life). Quality assessment addressed study design appropriateness, sample adequacy, follow-up completeness, tool validity, confounder control, and reporting transparency. Limitations including single-center design, cross-sectional nature, lack of ADPKD-specific cut-offs, and selection bias were critically noted.

### 2.4. Data Synthesis and Presentation

Given methodological heterogeneity, narrative synthesis grouped studies thematically: prevalence and determinants of malnutrition; organ volume–nutrition associations; nutritional impact on renal outcomes; pathophysiological mechanisms; and clinical implications. Findings were summarized descriptively, highlighting convergent evidence and knowledge gaps.

## 3. Evidence for Malnutrition and Sarcopenia in Adult ADPKD

Several key studies have systematically investigated the prevalence, determinants, and clinical significance of malnutrition and altered body composition in ADPKD [22] (Table 1). These studies provide convergent evidence for the importance of nutritional assessment in this population.

### 3.1. Organ Volume and Nutritional Status: The htTKLV Signal

Ryu et al. evaluated 288 ambulatory adult ADPKD patients and assessed the nutritional status using the modified Subjective Global Assessment (SGA), a validated semi-quantitative clinical tool that combines recent weight change, dietary intake, gastrointestinal symptoms, functional capacity and physical examination of muscle and subcutaneous fat stores into an overall score of nutritional risk. They quantified height-adjusted total kidney and liver volume (htTKLV) using CT [23].

Approximately 30% of patients were classified as at risk of malnutrition or malnourished by SGA, despite being managed as outpatients.

htTKLV was the strongest independent predictor of malnutrition: patients in the highest htTKLV category (cut-off ≈ 2340 mL/m) had ~8–9-fold higher odds of malnutrition compared with those below the cut-off, after adjusting for age, hemoglobin and eGFR.

eGFR, by contrast, was a weaker correlate of malnutrition once organ volume was included in multivariable models.

These data clearly show that, in ADPKD, intra-abdominal organomegaly (kidney + liver), rather than renal function per se, is the dominant correlate of nutritional risk in ambulatory patients (Figure 1).

### 3.2. Segmental BIA and the Body Composition Phenotype

In a subsequent study, Ryu et al. explored segmental bioelectrical impedance analysis (BIA) as a more objective tool to characterize body composition and fluid distribution [24]. Their analysis demonstrated that, compared with healthy controls, ADPKD patients had significantly higher total body water and elevated extracellular water/total body water (ECW/TBW), especially in the trunk and lower extremities. Several BIA-derived parameters correlated with SGA-defined malnutrition. In particular, ECW/TBW (whole body, trunk, lower extremities) > 0.38 and a reduced phase angle, a bioimpedance-derived index reflecting cell membrane integrity and body cell mass, with lower values indicating a poorer nutritional and muscular status, were associated with worse SGA categories. ECW/TBW in the trunk showed the strongest correlation with ln-htTKLV, directly linking organomegaly with altered fluid distribution. Collectively, these results define a characteristic ADPKD malnutrition phenotype: expansion of extracellular water, particularly in the trunk (where cystic organs are located), and reduced phase angle consistent with loss of cell mass and early sarcopenia. Notably, these changes were already present at early CKD stages, suggesting that they are not merely late uremic phenomena.

### 3.3. Confirmation from a More Recent Cohort: Early Sarcopenia

The 2025 J Nephrology study “Body water distribution, early malnutrition and sarcopenia in ADPKD” substantially extends these findings by embedding ADPKD within a chronic kidney disease (CKD) context rather than against healthy controls [25]. In this cross-sectional analysis, 218 CKD patients were enrolled: 71 with ADPKD and 147 with non-ADPKD CKD (stages 1–5D). After 1:1 propensity score matching, two balanced groups of 71 ADPKD and 71 non-ADPKD CKD patients were compared. All participants underwent BIA to quantify body water compartments, fat mass, fat-free mass, skeletal muscle mass and phase angle. Within the ADPKD arm, patients were further stratified according to organomegaly.

After matching, ADPKD patients still exhibited higher total and extracellular water than non-ADPKD CKD controls, both as absolute values (normalized to height squared) and as ECW/TBW, indicating that the fluid distribution abnormalities seen in earlier cohorts are not simply a generic CKD effect. Among ADPKD patients, those with organomegaly had lower phase angle, lower fat-free and skeletal muscle mass, and even lower fat mass than those without organomegaly, despite a paradoxically higher BMI, and these differences remained independent of kidney function. Notably, the mean phase angle observed in organomegalic ADPKD patients was in the range typically reported in frail geriatric or oncologic populations, underscoring the severity of their underlying protein–energy deficit despite an apparently “well-nourished” body habitus. BIA-derived abnormalities aligned with clinical indicators of nutritional risk, supporting their interpretation as early malnutrition and sarcopenia rather than mere overhydration. By directly comparing ADPKD with propensity-matched non-ADPKD CKD, this study shows that altered body water distribution and early sarcopenia are distinctive, organ volume-linked features of ADPKD, not confined to ESKD, dialysis or pre-transplant settings and not fully explained by CKD-related wasting alone.

### 3.4. Association with Renal Outcomes

While the cross-sectional work by Ryu et al. primarily established how frequent malnutrition is in ambulatory ADPKD [23] and how strongly it is driven by htTKLV, the prospective study by Lee et al. adds a crucial dimension by showing that the baseline SGA-defined nutritional status independently predicts short-term eGFR decline, thereby supporting malnutrition as a potential modifier rather than a mere correlate of disease severity [26]. In this multicenter cohort, 805 ambulatory adults with ADPKD were evaluated and 236 patients with “typical” ADPKD, imaging confirmation and a complete 1-year follow-up were included in the main analysis after exclusion of pediatric patients, individuals with ESKD and atypical imaging patterns. Their nutritional status at baseline was assessed by the Subjective Global Assessment (SGA; 7 = well nourished; 3–6 = at risk/malnourished), and the primary endpoint was an eGFR decline > 3 mL/min/1.73 m^2^ over one year, with additional secondary endpoints based on smaller eGFR declines and changes in proteinuria. Using multivariable logistic regression and propensity score matching to adjust for baseline eGFR, proteinuria, Mayo imaging class and other covariates, the authors showed that worse SGA categories were independently associated with a higher risk of rapid eGFR decline, with 38.6% of patients experiencing the primary endpoint over 12 months. Importantly, the association between a poorer nutritional status and faster renal function loss remained robust across sensitivity analyses and appeared independent of baseline proteinuria and imaging risk class, indicating that SGA captures prognostically relevant information not fully encoded by structural severity or classical CKD risk markers. Taken together with the strong links between organomegaly, BIA-derived markers of sarcopenia and malnutrition risk, these data reinforce the concept that malnutrition in ADPKD carries genuine prognostic significance and should be regarded as a clinically relevant, potentially modifiable component of disease management rather than a late or incidental complication.

### 3.5. Adiposity, Anthropometrics and the “Sarcopenic Obesity” Phenotype

Afsar et al. provide a narrative synthesis on adiposity and anthropometric measures in ADPKD [27]. While their primary emphasis is on obesity-related cardiovascular and renal risk, their conclusions are highly relevant to the malnutrition discussion. Across multiple cohorts, higher BMI, waist circumference and visceral adiposity are consistently associated with larger TKV, faster eGFR decline and greater symptom burden (pain, early satiety, dyspnea) in ADPKD. Proposed mechanisms include glomerular hyperfiltration, insulin resistance, adipokine imbalance and low-grade inflammation, all of which may promote cyst expansion, interstitial fibrosis and vascular injury.

Crucially, the authors explicitly highlight the risk of sarcopenic obesity, in which high fat mass and a large abdominal volume, driven by both visceral fat and cystic organs, coexist with low skeletal muscle mass and a poor functional status. In this setting, conventional anthropometric indices (BMI, waist circumference) become difficult to interpret, because they do not distinguish between metabolically active muscle, metabolically harmful visceral fat and metabolically “inert” cystic mass.

From a nutritional standpoint, this implies that “overweight” or “obese” ADPKD patients are not necessarily nutritionally protected; on the contrary, they may carry the double burden of adiposity-driven disease progression and organomegaly-related sarcopenia. Weight loss interventions, especially in patients with high BMI and large abdominal volume, may be desirable for both cardiovascular and renal outcomes, but they need to be carefully designed to be protein-sparing and muscle-preserving, so as to avoid unintentional exacerbation of protein–energy wasting. In practice, Afsar et al.’s analysis reinforces the idea that anthropometry alone is insufficient in ADPKD and must be complemented by structured nutritional assessment and body composition analysis to correctly identify patients who are simultaneously obese and malnourished.

Recognition of malnutrition and altered body composition in ADPKD necessitates practical strategies for assessment and intervention. The following section outlines evidence-based approaches to nutritional management in this population.

## 4. Pathophysiology Mechanisms Linking Organomegaly, Malnutrition and Renal Trajectory

Across imaging-, BIA- and clinically based studies, a coherent picture emerges: adults with ADPKD, particularly those with marked kidney and/or liver enlargement, display a distinctive, disease-specific alteration in body composition and fluid distribution [23,24,25,26,27]. Total body water and extracellular water are systematically increased, but without a proportional expansion of intracellular water, indicating predominantly local, cyst-related fluid accumulation rather than generalized volume overload. Because bioimpedance-derived lean mass is driven mainly by the intracellular compartment, this pattern suggests that bioelectrical impedance analysis is less reliable as a pure hydration tool in ADPKD, yet remains a valid and sensitive method for assessing the nutritional status and detecting early malnutrition and sarcopenia in this population.

These findings fit well with what could be termed the “body phenotype” of adult ADPKD [20]: a protuberant abdomen, driven by the combined effect of visceral adiposity and large cystic organs, sitting on progressively thinner extremities and declining skeletal muscle mass. In this context, BMI and crude body weight become profoundly misleading: organomegalic ADPKD patients often appear overweight or even obese, while in fact they have reduced fat-free mass, lower skeletal muscle indices and a phase angle in the range typically observed in geriatric or oncologic populations. A substantial subset of patients is therefore at a clinically relevant risk of protein–energy malnutrition and sarcopenia, yet remains under-recognized if assessment relies solely on conventional anthropometry and laboratory surrogates. Taken together, these data strongly support the concept that organomegaly itself represents a major and independent risk factor for malnutrition, potentially exerting a greater impact than CKD per se, while eGFR and BMI alone are poor surrogates for nutritional risk in ADPKD.

Several mechanisms are likely to converge to produce this phenotype. The mass effect of enlarged kidneys and liver promotes early satiety, anorexia and gastrointestinal discomfort, chronically limiting energy and protein intake and discouraging complete meals. In addition, marked abdominal distension and visible organomegaly impose a persistent psychological burden, with body image distortion, embarrassment and social withdrawal, which may further blunt appetite and motivation to prepare balanced meals and to engage in physical activity [28,29].

In parallel, systemic inflammation, insulin resistance, increased metabolic demands and high glucose uptake by proliferating cyst-lining cells, consistent with a form of glycolytic reprogramming reminiscent of cancer metabolism, may further tilt the balance towards catabolism and loss of muscle protein [30,31,32,33,34]. These disease-specific alterations are superimposed on the more “classical” CKD drivers of protein–energy wasting, metabolic acidosis, endocrine disturbances, anemia and reduced physical activity, creating a chronic, low-grade catabolic milieu even in earlier stages of renal impairment [35].

The net result is a scenario in which organomegalic ADPKD patients accumulate cystic fluid and extracellular water, but progressively lose muscle mass and physiological reserve, often at a stage when eGFR is still moderately preserved and overt cachexia is not clinically suspected. Clinically, this may translate into disproportionate fatigue, reduced exercise tolerance, increased vulnerability to intercurrent illness and hospitalization, and a lower threshold for functional decline after acute stressors, despite apparently “stable” kidney function and a BMI that remains in the overweight or obese range. From a clinical standpoint, these observations argue that malnutrition and sarcopenia should be regarded as central, disease-modifying components of the ADPKD phenotype, rather than late, incidental complications confined to dialysis and pre-transplant settings. Routine, structured nutritional assessment (e.g., SGA/PG-SGA), complemented where available by BIA-based body composition analysis, should be integrated into ADPKD care pathways, with particular attention to patients with severe organomegaly, symptomatic polycystic liver disease and the “typical” ADPKD body phenotype. In such patients, the finding of a low phase angle or reduced muscle indices despite “normal” or high BMI should prompt early, proactive intervention rather than reassurance.

At the molecular level, ADPKD is characterized by dysregulation of multiple pathways that may directly contribute to malnutrition and sarcopenia. Aberrant mTOR (mechanistic target of rapamycin) signaling—a hallmark of cyst growth—also plays a central role in muscle protein synthesis and metabolic homeostasis [18].

Paradoxically, while mTOR is hyperactivated in cyst-lining epithelial cells, systemic or skeletal muscle mTOR activity may be suppressed in the context of chronic inflammation and metabolic stress, potentially contributing to muscle wasting [18,19]. Chronic low-grade inflammation, evidenced by elevated circulating cytokines (IL-6, TNF-α, CRP) even in early-stage ADPKD [19], can promote muscle catabolism and impair appetite regulation through multiple molecular mechanisms including NF-κB activation, ubiquitin–proteasome system upregulation, and inhibition of anabolic pathways [36,37,38]. Additionally, altered insulin signaling and mitochondrial dysfunction—both reported in ADPKD [19]—may compromise energy metabolism and muscle protein synthesis. These molecular disturbances, combined with mechanical and nutritional factors, create a complex milieu favoring protein–energy wasting and sarcopenia [39].

## 5. Clinical Implications and Practical Approach

The convergent evidence presented argues for integrating systematic nutritional assessment into routine ADPKD care well before advanced CKD or dialysis. High-priority candidates include patients with marked organomegaly, the characteristic body phenotype (protuberant abdomen with relatively thin extremities), early satiety, unintentional weight loss, or accelerated eGFR decline despite apparently normal BMI. Early recognition of this “hidden malnutrition” may identify a modifiable contributor to disease progression and adverse outcomes. Table 2 outlines a pragmatic framework combining validated screening tools (SGA, PG-SGA), basic anthropometry (BMI, waist and mid-arm circumferences), functional measures (handgrip strength), and—where available—bioelectrical impedance analysis.

In ADPKD, BIA-derived parameters (ECW/TBW ratio, phase angle, skeletal muscle estimates) should be interpreted primarily as body composition indicators rather than volume status guides, given the characteristic pattern of localized extracellular fluid expansion within cystic organs. Concurrently, emerging interest in dietary interventions targeting metabolic pathways—including caloric restriction, intermittent fasting, time-restricted feeding, and ketogenic regimens—has generated preliminary evidence suggesting potential benefits for cyst growth attenuation through mTOR inhibition and metabolic reprogramming [40]. Early-phase trials (RESET-PKD, KETO-ADPKD) demonstrate feasibility and hint at structural or metabolic improvements in selected endpoints [41,42]. However, long-term renal and safety outcomes remain uncertain, and no specific dietary pattern is yet endorsed as disease-modifying therapy in consensus guidelines [43]. These experimental approaches warrant cautious interpretation as investigational adjuncts requiring specialist oversight, particularly given risks of exacerbating protein–energy wasting, nephrolithiasis, and cardiovascular complications in vulnerable subgroups. Critically, ketogenic or severely carbohydrate-restricted diets, while potentially beneficial in well-nourished patients with rapid progression, pose substantial hazards in those with pre-existing malnutrition or sarcopenia. Such restrictive regimens demand meticulous monitoring to prevent protein depletion and micronutrient deficiencies. Current evidence supports selective, rather than routine, application under nephrology and dietetics guidance, with priority given to restoring nutritional adequacy before implementing restrictive dietary strategies. Ultimately, the therapeutic objective is a precision medicine approach: preserve or restore lean mass, avoid unnecessary restrictions, and judiciously manage adiposity. Whether correcting organomegaly-driven malnutrition translates into improved quality of life, reduced complications, and slower renal decline requires rigorous prospective validation. Integrating nutritional optimization into multidisciplinary ADPKD care—alongside hypertension management, disease-modifying pharmacotherapy, and surveillance for extrarenal manifestations—represents an actionable, evidence-informed strategy while awaiting definitive interventional trials on metabolic and dietary modulation.

## 6. Conclusions and Future Perspectives

This review synthesizes accumulating evidence that malnutrition and sarcopenia represent clinically significant yet under-recognized complications of adult ADPKD, driven primarily by progressive organomegaly rather than kidney function decline alone. Patients with marked renal and hepatic enlargement exhibit altered body composition—reduced muscle mass, elevated extracellular water, depressed phase angle—despite frequently normal or elevated BMI. Critically, these nutritional disturbances manifest even with preserved eGFR, challenging conventional assumptions that protein–energy wasting is confined to advanced CKD.

**Figure 2:**
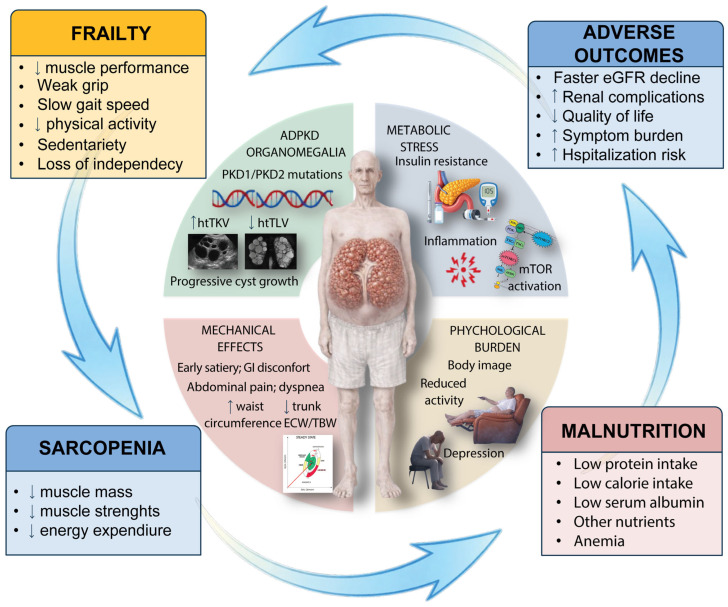
Pathophysiological pathway linking ADPKD organomegaly to malnutrition, sarcopenia, and adverse outcomes. The circular diagram illustrates how progressive organomegaly drives malnutrition and sarcopenia through three main mechanisms: mechanical compression (early satiety, reduced oral intake), metabolic dysregulation (mTOR pathway dysregulation, insulin resistance), and chronic inflammation (elevated cytokines promoting catabolism). This cascade leads to frailty, functional decline, and adverse clinical outcomes including hospitalizations, reduced quality of life, and accelerated renal function decline.

The pathophysiological substrate is multifactorial: it covers mechanical compression producing early satiety and reduced intake; metabolic derangements encompassing chronic inflammation, mTOR dysregulation, and insulin resistance favoring catabolism; and psychological burdens from body image disturbance and activity restriction. Figure 2 illustrates this integrative pathway linking organomegaly to malnutrition, sarcopenia, and adverse outcomes.

Prospective data demonstrate that the baseline nutritional status independently predicts eGFR trajectory after adjustment for conventional risk factors, suggesting malnutrition may actively contribute to progression rather than merely reflecting disease severity. The paradoxical coexistence of obesity with sarcopenia—“sarcopenic obesity”—underscores BMI’s inadequacy as a standalone metric and highlights the necessity of body composition assessment.

From a clinical standpoint, systematic nutritional screening is warranted, particularly in patients with substantial organomegaly or accelerated functional decline. Validated tools (SGA, MUST) combined with bioelectrical impedance can identify at-risk individuals and quantify body composition disturbances independent of cyst burden. Early dietetic intervention emphasizing protein adequacy and resistance exercise may preserve lean mass, though caution is required with restrictive regimens (e.g., ketogenic diets) that risk exacerbating existing deficits.

Critical knowledge gaps persist. First, prospective trials testing whether nutritional optimization improves hard outcomes—progression rates, hospitalization, quality of life—are lacking. Second, ADPKD-specific thresholds for malnutrition biomarkers remain undefined, limiting cross-study generalizability. Third, the relative contributions of mechanical, metabolic, and psychological drivers in individual patients require clarification to enable precision targeting. Finally, long-term safety and efficacy data for dietary interventions spanning the spectrum from protein supplementation to caloric restriction are needed.

Future research priorities include randomized controlled trials of nutritional interventions with clinically meaningful endpoints (eGFR trajectory, hospitalization, patient-reported outcomes), integration of body composition assessment into disease-modifying therapy trials (e.g., tolvaptan), mechanistic investigations of the organomegaly–inflammation–sarcopenia axis, and development of validated ADPKD-specific nutritional risk stratification tools.

In summary, malnutrition and sarcopenia constitute a “hidden iceberg” in ADPKD—masked by normal BMI yet contributing substantively to disease burden. Systematic screening, objective body composition analysis, and targeted interventions represent an actionable frontier in comprehensive care that, when integrated with established management pillars (blood pressure control, disease-modifying therapy, extrarenal surveillance), may improve outcomes for patients navigating this complex lifelong disorder.

## Figures and Tables

**Table 1:** Key studies addressing malnutrition, body composition and outcomes in adult ADPKD.

Study	Design/Setting	Population	Main Tools/Exposures	Main Nutritional Body Composition Findings	Key Message
Ryu et al. [23]	Cross-sectional cohort	288 ambulatory adults with ADPKD	Modified SGA; CT-based htTKLV	~30% at risk of or malnourished; htTKLV is the strongest independent predictor of malnutrition; eGFR is a weaker correlate	In ambulatory ADPKD, intra-abdominal organomegaly (kidney + liver), rather than eGFR, drives malnutrition risk.
Ryu et al. [24]	Cross-sectional, same cohort as above	288 ADPKD vs. healthy controls	Segmental BIA; htTKLV; SGA	ADPKD: ↑ TBW and ↑ ECW/TBW (trunk, legs); ↓ phase angle associated with worse SGA; trunk ECW/TBW strongly correlated with htTKLV	Defines an ADPKD-specific BIA phenotype linking organomegaly, ECW expansion and reduced phase angle to early sarcopenia.
Sciarrone Alibrandi et al. [25]	Cross-sectional, matched CKD cohort	71 ADPKD vs. 147 non-ADPKD CKD (stages 1–5D); 1:1 matched	Whole-body BIA; organomegaly by MRI/US	ADPKD: ↑ TBW and ↑ ECW/TBW vs. non-ADPKD CKD; within ADPKD, organomegaly → ↓ phase angle, ↓ FFM/SMM and ↓ fat mass despite ↑ BMI	Early sarcopenia and altered body water distribution are distinctive, organ volume-linked features specific to ADPKD.
Lee et al. [26]	Multicenter prospective cohort	236 “typical” ADPKD with 1-year follow-up	SGA (7 vs. 3–6); clinical data; imaging class	Baseline SGA 3–6 (at risk/malnourished) associated with higher odds of eGFR decline > 3 mL/min/1.73 m^2^ over 1 year	Nutritional status independently predicts short-term eGFR decline; malnutrition is a potential modifier of renal trajectory.
Afsar et al. [27]	Narrative review	N/A (review)	Anthropometry; adiposity; metabolic factors	Higher BMI, waist and visceral fat associated with larger TKV, faster eGFR loss and more symptoms; concern for sarcopenic obesity	Overweight ADPKD patients may be simultaneously obese and sarcopenic; anthropometry alone is insufficient.
Khan et al. [22]	Narrative review, PLD	PLD with or without ADPKD	Nutritional guidelines; clinical data	PLD: early satiety, reduced intake, high malnutrition and sarcopenia risk; recommends systematic screening and tailored diet	PLD provides a mechanistic template for organomegaly-driven malnutrition relevant to ADPKD with severe liver involvement.

**Table 2:** Suggested practical approach to nutritional assessment and management in adult ADPKD.

Domain	Suggested Approach
Patients at higher nutritional risk	Marked kidney and/or liver enlargement (high htTKV/htTKLV, advanced Mayo class) Symptomatic PLD (early satiety, postprandial discomfort, abdominal pain, dyspnea) Rapid eGFR decline despite “normal” or high BMI Typical ADPKD “body phenotype” (protuberant abdomen with relatively thin extremities)
Baseline screening tools	SGA or PG-SGA (global clinical assessment of nutritional status) Weight, BMI, waist circumference, mid-arm circumference Simple functional tests (e.g., handgrip strength, reported physical performance)
Extended assessment (where available)	Bioelectrical impedance analysis: ECW/TBW, phase angle, fat-free mass, skeletal muscle mass Opportunistic assessment of muscle area/density on CT/MRI performed for TKV/PLD (e.g., psoas or paraspinal muscle indices)
Red flags/concerning patterns	SGA indicating malnutrition risk or frank malnutrition Elevated ECW/TBW and/or disproportionate trunk ECW/TBW Low phase angle for age/sex (or clearly lower in organomegalic vs. non-organomegalic patients) Reduced skeletal muscle indices despite overweight/obesity Unintentional weight loss in the context of progressive organomegaly
Management principles	Preserve or restore lean mass: adequate protein and energy intake tailored to CKD stage In PLD with early satiety: small, frequent, energy- and protein-dense meals; avoid unnecessary dietary restrictions In overweight/obese ADPKD: gradual, supervised weight loss with protein-sparing strategies and resistance exercise to prevent protein–energy wasting Regarding ketogenic or very-low-carbohydrate diets: exercise caution, particularly in patients with existing malnutrition; such interventions require close monitoring and are not universally recommended
Integration into ADPKD care	Incorporate nutritional screening into routine visits Interpret BMI and weight in the context of organ volume and body composition analysis, rather than in isolation Reassess nutritional status during major changes in treatment or disease trajectory

## Data Availability

No new data were created or analyzed in this study.

## Abbreviations

The following abbreviations are used in this manuscript: ADPKD, autosomal dominant polycystic kidney disease; BIA, bioelectrical impedance analysis; BMI, body mass index; CKD, chronic kidney disease; CRP, C-reactive protein; CT, computed tomography; ECW, extracellular water; eGFR, estimated glomerular filtration rate; ESKD, end-stage kidney disease; GI, gastrointestinal; htTKLV, height-adjusted total kidney and liver volume; htTKV, height-adjusted total kidney volume; ICW, intracellular water; IL-6, interleukin-6; KDOQI, Kidney Disease Outcomes Quality Initiative; MRI, magnetic resonance imaging; mTOR, mechanistic target of rapamycin; MUST, Malnutrition Universal Screening Tool; PhA, phase angle; PG-SGA, Patient-Generated Subjective Global Assessment; PKD1/PKD2, polycystic kidney disease genes 1 and 2; PLD, polycystic liver disease; RCT, randomized controlled trial; SGA, Subjective Global Assessment; TBW, total body water; TKV, total kidney volume; TNF-α, tumor necrosis factor alpha.
